# 'Who's who' in two different flower types of *Calluna vulgaris *(*Ericaceae*): morphological and molecular analyses of flower organ identity

**DOI:** 10.1186/1471-2229-9-148

**Published:** 2009-12-14

**Authors:** Thomas Borchert, Katrin Eckardt, Jörg Fuchs, Katja Krüger, Annette Hohe

**Affiliations:** 1 Institute of Vegetable and Ornamental Crops (IGZ), Department Plant Propagation, Kuehnhaeuser Str 101, 99189 Erfurt, Germany; 2 University of Applied Sciences Dresden, Faculty for Agriculture and Landscape Management, Pillnitzer Platz 2, 01326 Dresden, Germany; 3 Leibniz-Institute of Plant Genetics and Crop Plant Research (IPK), Department of Cytogenetics and Genome Analysis, Corrensstrasse 3, 06466 Gatersleben, Germany

## Abstract

**Background:**

The ornamental crop *Calluna vulgaris *is of increasing importance to the horticultural industry in the northern hemisphere due to a flower organ mutation: the flowers of the 'bud-flowering' phenotype remain closed i.e. as buds throughout the total flowering period and thereby maintain more colorful flowers for a longer period of time than the wild-type. This feature is accompanied and presumably caused by the complete lack of stamens. Descriptions of this botanical particularity are inconsistent and partially conflicting. In order to clarify basic questions of flower organ identity in general and stamen loss in detail, a study of the wild-type and the 'bud-flowering' flower type of *C. vulgaris *was initiated.

**Results:**

Flowers were examined by macro- and microscopic techniques. Organ development was investigated comparatively in both the wild-type and the 'bud-flowering' type by histological analyses. Analysis of epidermal cell surface structure of vegetative tissues and perianth organs using scanning electron microscopy revealed that in wild-type flowers the outer whorls of colored organs may be identified as sepals, while the inner ones may be identified as petals. In the 'bud-flowering' type, two whorls of sepals are directly followed by the gynoecium. Both, petals and stamens, are completely missing in this flower type. The uppermost whorl of green leaves represents bracts in both flower types.

In addition, two MADS-box genes (homologs of *AP3/DEF *and *SEP1/2*) were identified in *C. vulgaris *using RACE-PCR. Expression analysis by qRT-PCR was conducted for both genes in leaves, bracts, sepals and petals. These experiments revealed an expression pattern supporting the organ classification based on morphological characteristics.

**Conclusions:**

Organ identity in both wild-type and 'bud-flowering' *C. vulgaris *was clarified using a combination of microscopic and molecular methods. Our results for bract, sepal and petal organ identity are supported by the 'ABCDE model'. However, loss of stamens in the 'bud-flowering' phenotype is an exceptional flower organ modification that cannot be explained by modified spatial expression of known organ identity genes.

## Background

**Figure 1:**
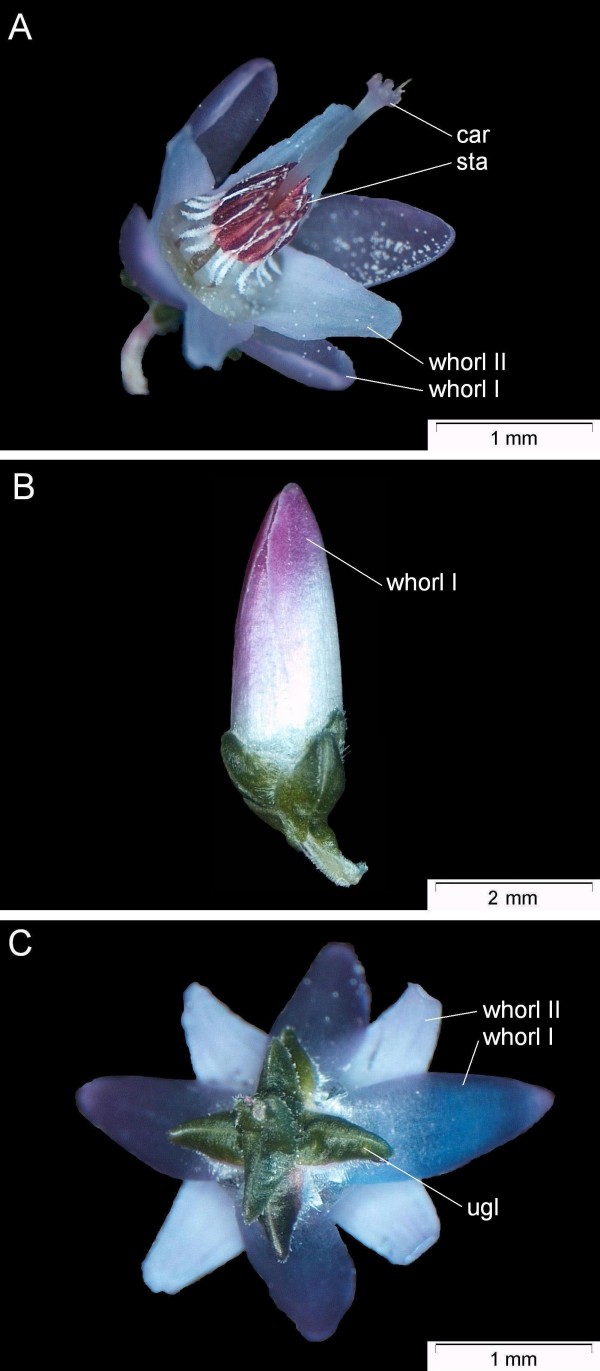
**Flower types of *C. vulgaris***. A: wild-type (*Niederohe *from Lueneburger Heide, Germany). B: 'bud-flowering' ('Amethyst'); C: bottom of wild-type (*Niederohe *from Lueneburger Heide, Germany) flower; Labels are: car (carpels), sta (stamens), ugl (uppermost whorls of green leaves). The bipartites perianth is separated in whorl I and whorl II organs.

*Calluna vulgaris *L. (Hull.) (Fig. 1A) belongs to the order *Ericales*, which comprises 25 families including 346 genera with more than 11,500 species in total [1]. The *Ericales *incorporate about 5.9% of core eudicot diversity, one third of which is made up of the *Ericaceae *alone [1]. The economic significance of *C. vulgaris *to the horticultural industry in Europe and North-America is continually increasing [2]. The current market share in Germany for instance, amounts to approximately 141 million EUR, or > 100 million plants per year, respectively [2]. In principal, this economic significance is the results of a single but considerable change in the flower morphology: the loss of stamens that is accompanied by a non-opening of the flower bud. In contrast to wild-type flowers (Fig. 1A) that are only attractive from August to October the resulting 'bud-flowering' phenotype (Fig. 1B) preserves its unpollinated stigmas within the never-opening buds and has an extended flowering period up to December. For this reason, it is the most valuable flower type of this species to the horticultural business. In contrast, other forms, such as the 'filled' or the 'multi-bracteate' types are less important. Previous investigations revealed the monogenic recessive inheritance of the 'bud-flowering' trait [3] that was described in literature for the first time (as far as known by the authors) in 1935 [4].

The synoecious flower of wild-type *C. vulgaris *is of radial symmetry, posing with two outer perianth whorls with four to five colored organs in each whorl, two whorls of four to five stamens and four to five coadunate carpels [5-7]. The sepals are grouped into two distinct whorls of two times two [8]. The colored organs of the perianth whorl II are fused at the receptacle to form a corolla tube [5,9]. Two whorls of at least six green leaves in total surround the flower [10]. These uppermost whorls of green leaves do not match the perianth symmetry, since they are aligned with the sepal whorl instead with the petal whorl (Fig. 1C: indicated as ugl).

In contrast, the 'bud-flowering' type completely lacks the male reproductive organs, which is probably at least one of the reasons for its developmental arrest in the bud stage. In 1986, three different subforms of the 'bud-flowering' type were described [[11], page 281]: *f. diplocalyx *(' [...] eight instead of four sepals and usually neither stamens nor corolla [...]'), *f. polysepala *(' [...] similar to *f. diplocalyx *but [...] there are indeed many sepals, more than eight.') and *f. clistanthes *(' [...] flower parts are present in the normal number, but the corolla never, or hardly, opens.'). Evidences or justifications for this classification of organs e.g. of the colored organs either as sepals or petals are absent [5,11]. Moreover, no explanation is given for the grouping of the sepals into two whorls and for the grouping of stamen in two whorls [5,8]. Furthermore, the described classification of *f. polysepala *and *f. clisthantes *could not be reproduced by the authors, since the cultivars that are given as examples all looked like the *diplocalyx*-type in our hands.

Two different approaches are commonly applied to identify organ characteristics in the perianth of angiosperms: morphological comparisons and gene expression studies [12]. The molecular procedure mainly investigates the expression of the floral homeotic genes. According to the classical 'ABCDE'-model of flower organ identity, changes in flower morphology are the results of expression shifts of different classes of floral homeotic genes encoding transcription factors in the corresponding whorls (see, e.g. [[13,14] or [15]]): class A gene function in the outmost whorl leads to the formation of sepals; combined expression of class A and B genes in the second whorl leads to the formation of petals; class B and C gene function in whorl three promotes the development of stamens, and expression of class C genes in the innermost whorl leads to the development of carpels. Additionally, class D gene function is required for ovule formation, whereas class E gene function is required for the development of all organs, respectively (see. e.g. [16-19]). Several studies demonstrated that the perianth organs can be distinguished by the assessment of their epidermal cell surface structure by scanning electron microscopy (SEM), as shown in *Arabidopsis thaliana *[16] or in the *Ericales *(*Impatiens*, *Marcgravia*) [20]. Both assays - the morphological and the molecular assay - have to be regarded as complementary [12].

Regarding the indistinct descriptions and the lack of current in-depth studies and molecular data in *C. vulgaris*, several uncertainties still exist on the topic of the flower organ identity in this species. On the one hand, questions arise regarding the discrete identity of the two outer whorls of colored organs. On the other hand, the lack of the androecium in the 'bud-flowering' type has not been ascertained either. Until now, it is even uncertain, whether stamen development is been initiated or whether the initiation of primordia is inhibited.

The determination of the flower organ identity and the understanding of the development of the 'bud-flowering' mutation itself are of importance for future breeding efforts in *C. vulgaris *since the 'bud-flowering' phenotype is the most important breeding target in this species. We therefore initiated histological, microscopic and molecular examinations to clarify the identity of flower organs and of existent differences between wild-type and 'bud-flowering' phenotypes.

## Results

In order to elucidate the unknown organ identities of the two most important flower phenotypes in *C. vulgaris *flower development was monitored histologically for both, the wild-type as well as the 'bud-flowering' type. In addition, the perianth organs were examined by SEM and became successfully distinguishable among themselves and if compared to bracts and leaves. In order to achieve a better understanding of mutations in flower morphology in this crop, an initial cloning of two MADS-box genes was realized in addition to preliminary expression analyses. The genome size was determined in order to evaluate the chances of future cloning of new unknown genes by map-based cloning.

### Morphological perianth organ analysis of the wild-type and the 'bud-flowering' phenotype

**Figure 2:**
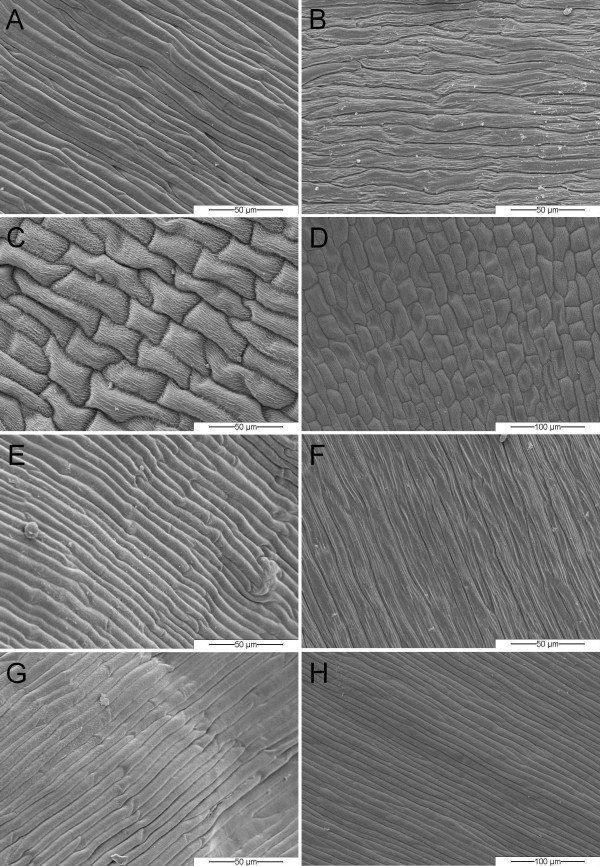
**Comparative SEM observations of abaxial and adaxial epidermal surface structures of *C. vulgaris *perianth organs**. wild-type whorl I, abaxial (A), adaxial (B); wild-type whorl II, abaxial (C), adaxial (D); 'bud-flowering' whorl I, abaxial (E), adaxial (F); 'bud-flowering' whorl II, abaxial (G), adaxial (H);

In wild-type phenotypes, the whorl II organs are commonly fused at their base and are more delicate compared to whorl I organs, which are clearly separated and appear quite robust. In contrast, 'bud-flowering' organs of whorl II are not fused and resemble the whorl I organs in shape, color and stability. SEM of the abaxial and adaxial epidermis structures (n = 4 varieties each) of whorl I and whorl II organs in both the wild-type and the 'bud-flowering' phenotype was carried out (Fig. 2) to identify, whether whorl I organs in the wild-type can be identified as sepals or petals and in order to clarify the identity of the whorl II organs in the 'bud-flowering' phenotype. Cells of the outermost whorls of the wild-type phenotype are flat and stretched (Fig. 2AB). In contrast, cells of the second whorl appear bloated ('dome-shaped'), are shorter in diameter and length and are striated with papillate structures (Fig. 2CD). On the contrary, the cell surfaces of the 'bud-flowering' perianth organs are indistinguishable from each other, since both whorls consist of the flat and stretched cell type (Fig. 2E-H), comparable to the outmost whorl of the wild-type. In particular, the second whorl leaves are not 'dome-shaped'. Thus, concerning whorl I organs of the wild-type phenotype, both their position and their cell surface structure indicate a sepaloid identity, whereas their color suggests a petaloid identity. Regarding whorl II organs, all three criteria investigated may be a hint to petaloid identity. In contrast, all organs in both perianth whorls of the 'bud-flowering' phenotypes are morphologically not distinguishable and show the same characteristics as whorl I organs of the wild-type phenotype. Therefore, they are likewise presumably to be identified as sepals by two out of the three criteria mentioned above; once more, their coloring suggests a petaloid identity.

**Figure 3:**
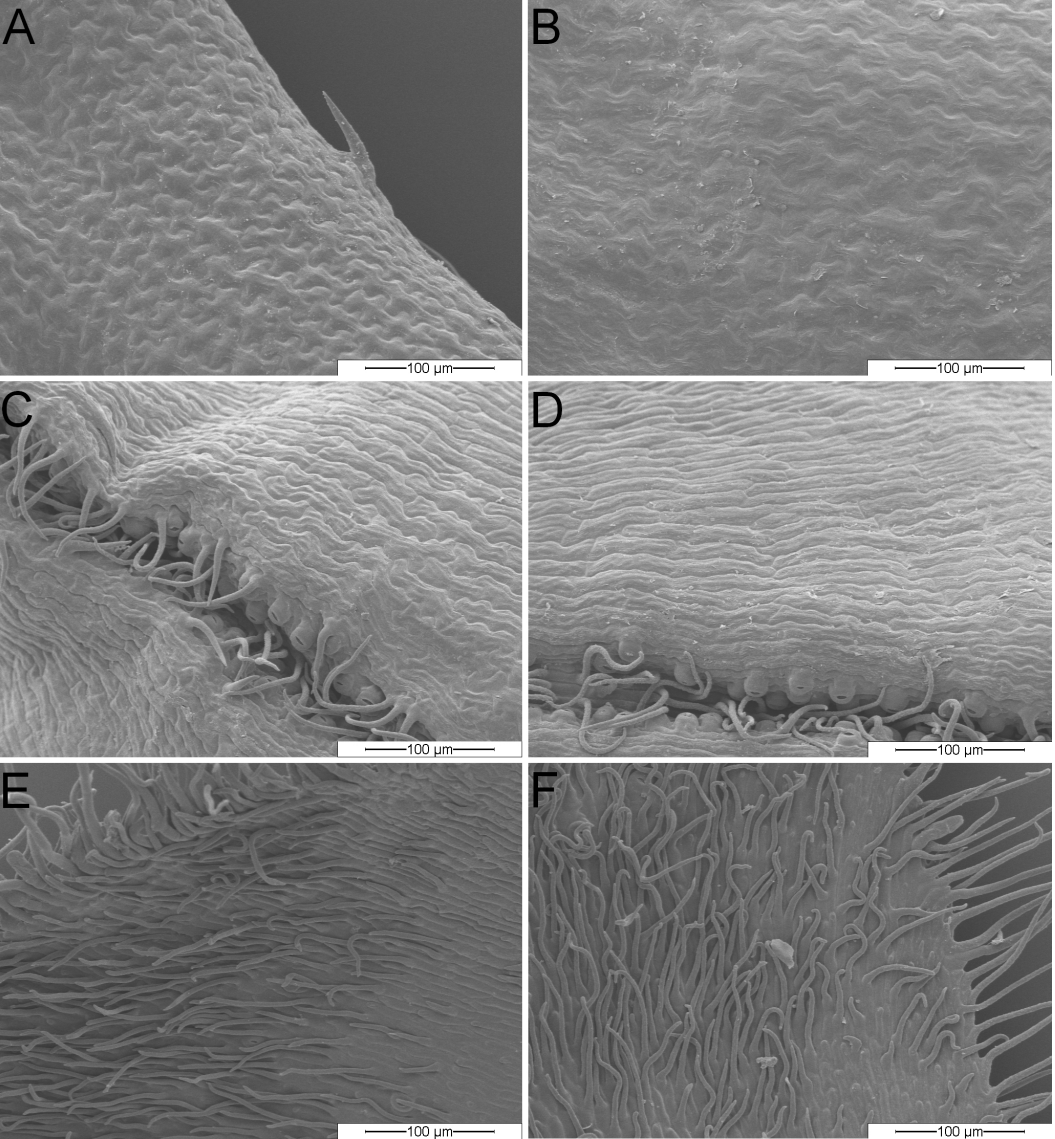
**Comparative SEM observations of abaxial and adaxial epidermal surface structures of *C. vulgaris *tissues**. wild-type leaf tissue (A), 'bud-flowering' leaf tissue (B); wild-type bracts, abaxial side (C), adaxial side (E); 'bud-flowering' bracts, abaxial side (D), adaxial side (F);

Differentiation between bracts and leaves by morphological characteristics became possible via SEM analysis of both tissues (Fig 3). The surface structure of leaf tissue of both flower types (Fig. 3AB) showed a puzzle-like cell structuring, both on ad- and abaxial sides. In contrast, in the uppermost whorls of green leaves of both flower types as indicated in Fig. 1C, we identified a slightly differing cell structure. The abaxial side (Fig. 3CD) is characterised by the occurrence of a channel, in which most of the stomata are located (Fig. 3CD), whereas the adaxial side is covered with hair-like structures (Fig. 3EF). Therefore, we assume these uppermost whorls of green leaves to be bracts. However, bracts and leaves resemble each other in the occurrence of stomata (not shown for leaves) which, in contrast, we did not observe in any colored perianth organ.

### Cloning of MADS-box genes

MADS-box transcription factors were identified using RACE-PCR. Our initial 3'-RACE experiments resulted in the cloning of two gene fragments, one putative *AP3/DEF*-like gene we named *CvAP3 *[Genbank:GQ202026], and one *SEP1/2*-like gene we named *CvSEP1 *[Genbank:GQ202027]. For *CvAP3*, the sequence data resulted from three independent experimental PCR and cloning assays. *CvSEP1 *was cloned by chance since the primer was originally designed to amplify B-genes. Thus, *CvSEP1 *could not be verified independently until now. Both partial genes were obtained by cloning a PCR fragment of approximately 950 bp.

Using the BLAST conserved domain database [21], the K-box and the (partial) MADS-box were identified in *CvAP3*, and the K-box in *CvSEP1*. Furthermore, both the EuAP3 motif and the PI derived motif [22] were identified within *CvAP3*, whereas the *CvSEP1 *gene included the SEP I and SEP II motif [23]. The latter motif, also termed as *AGL2/SEP1 *terminal motif [24], may be used to discriminate *SEP1/2 *(the *LOFSEP *clade) and *SEP3 *genes: *SEP3 *genes are missing this motif, but instead, they contain either the *AGL9/SEP3 *or the *ZmM7 *motif [24]. Our approach to furthermore determine gene homology by calculating phylogenetic similarities based on nucleotide alignments (Additional Files 1 and 2) resulted in unrooted phylograms (Additional Files 3 and 4) of sparse information content due to low posterior probability values for *C. vulgaris *samples. The connection of *CvAP3 *remains unresolved, since it is rather placed near the *Arabidopsis *outgroup than near any of the included genes of the *Ericales *family (*Primula*, *Marcgravia*, *Impatiens*). In case of *CvSEP1*, the *Calluna *gene is placed near *Diospyros kaki *which is, beneath *Impatiens*, the only available sample from the *Ericales*. In both cases, the anticipated outgroup genes are identifiable.

### Molecular perianth organ analysis of the wild-type and the 'bud-flowering' type

**Figure 4:**
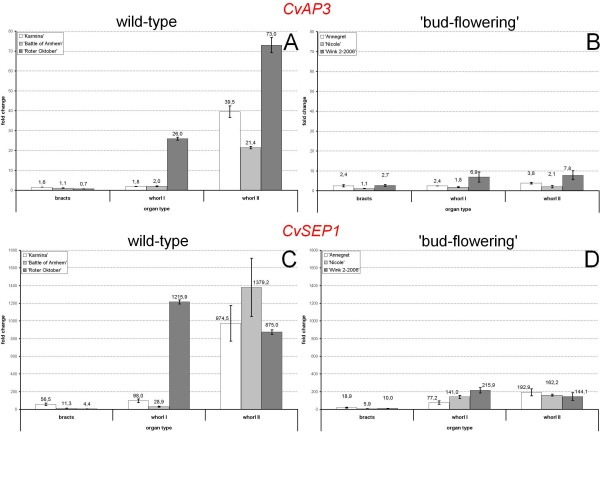
**Expression analysis of *CvAP3 *(A) and *CvSEP1 *(B) in *C. vulgaris *flower tissues**. Normalized (vs. 18S rRNA) expression is presented for both the wild-type and the 'bud-flowering' type as fold change (ΔΔCt) of arbitrary units vs. the reference tissue (leaf tissue).

The relative expression of the *C. vulgaris AP3/DEF*- and *SEP1/2*-like genes was analysed in three different genotypes per flower type (Fig. 4). ΔΔCt-values have been calculated to compare expression levels between the different flower tissues including bracts and the leaf tissue of the corresponding flower type, since the expression of both genes was lowest (albeit present, compared to the normalizer) in leaves. For better comparison between wild-type (Fig. 4AC) and 'bud-flowering' (Fig. 4BD) samples, the Y-axes are uniformly scaled for each gene.

Although the expression levels of both genes were genotype-specific, an overall organ-specific expression patterns were revealed. The expression levels of *CvAP3 *significantly increased in whorl II organs of the wild-type compared to whorl I organs and bracts in the three tested genotypes (Fig. 4A). Only 'Roter Oktober' showed an increased expression of *CvAP3 *in whorl I organs, too (albeit lower compared to whorl II). This petal-related increase of mRNA amount was not observed in all three 'bud-flowering' genotypes (Fig. 4B). These data support the morphological classification of whorl II organs in the 'bud-flowering' types to be a second whorl of sepals.

For *CvSEP1 *(Fig. 4CD), expression was higher in both perianth whorls in both flower types with an at least 1.7X increase of expression between bracts and whorl I organs. The variety 'Roter Oktober' showed an almost 1.4X higher expression of *CvSEP1 *in whorl I organs than in whorl II organs. In the 'bud-flowering' phenotypes, the expression of *CvSEP1 *did not differ markedly between whorl I and whorl II organs (except for 'Annegret', approx. 2.5X increase) and was clearly lower if compared to the corresponding wild-type organs, respectively. The differences of especially *CvSEP1 *gene expression between leaf tissue and the uppermost green leaves furthermore supports our morphology-based classification of the latter ones as bracts.

For both target genes, the unusual expression in leaf tissue was confirmed in several independent samples of different ages of three other wild-type and 'bud-flowering' genotypes. Cloning and sequencing of these PCR products confirmed the identity of the amplified transcripts.

### Floral formula of different flower types

Since we were not able to decide whether organs of the same identity were arranged in one or several whorls, we uniformly speak of one whorl per organ type, except for flower types with changes in organ identity. Thus, the floral formulas presented are based on the described morphological (e.g. cell surface structure) and molecular results and not on positional information of the organs.

**Figure 5:**
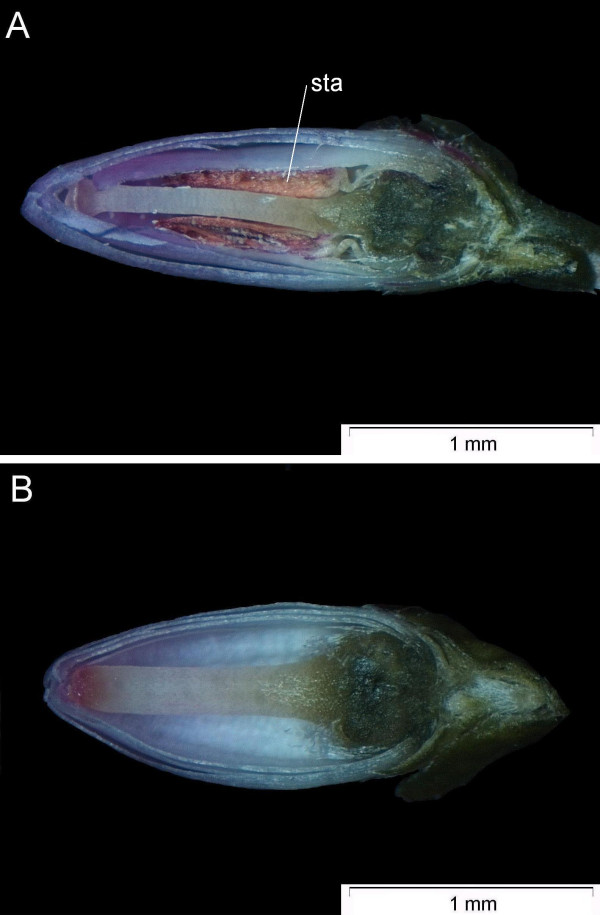
**Sagittal slices of mature flower buds**. A: wild-type phenotype (*Niederohe *from Lueneburger Heide, Germany): Ca^4^Co^(4)^A^8^G^(4)^; B: 'bud-flowering' phenotype ('Anneliese'): Ca^4+4^Co^0^A^0^G^(4)^; The label indicates stamens (sta) in the wild-type flower.

In contrast to the wild-type (Ca^4^Co^(4)^A^8^G^(4)^, Fig. 5A; Ca: calyx; Co: corolla; A: androecium; G; gynoecium), the 'bud-flowering' phenotype completely lacks stamens whereas its petals are transformed to sepals (Ca^4+4^Co^0^A^0^G^(4)^, Fig. 5B). This type corresponds to the 'diplocalyx' type [12].

### Flower organ development

**Figure 6:**
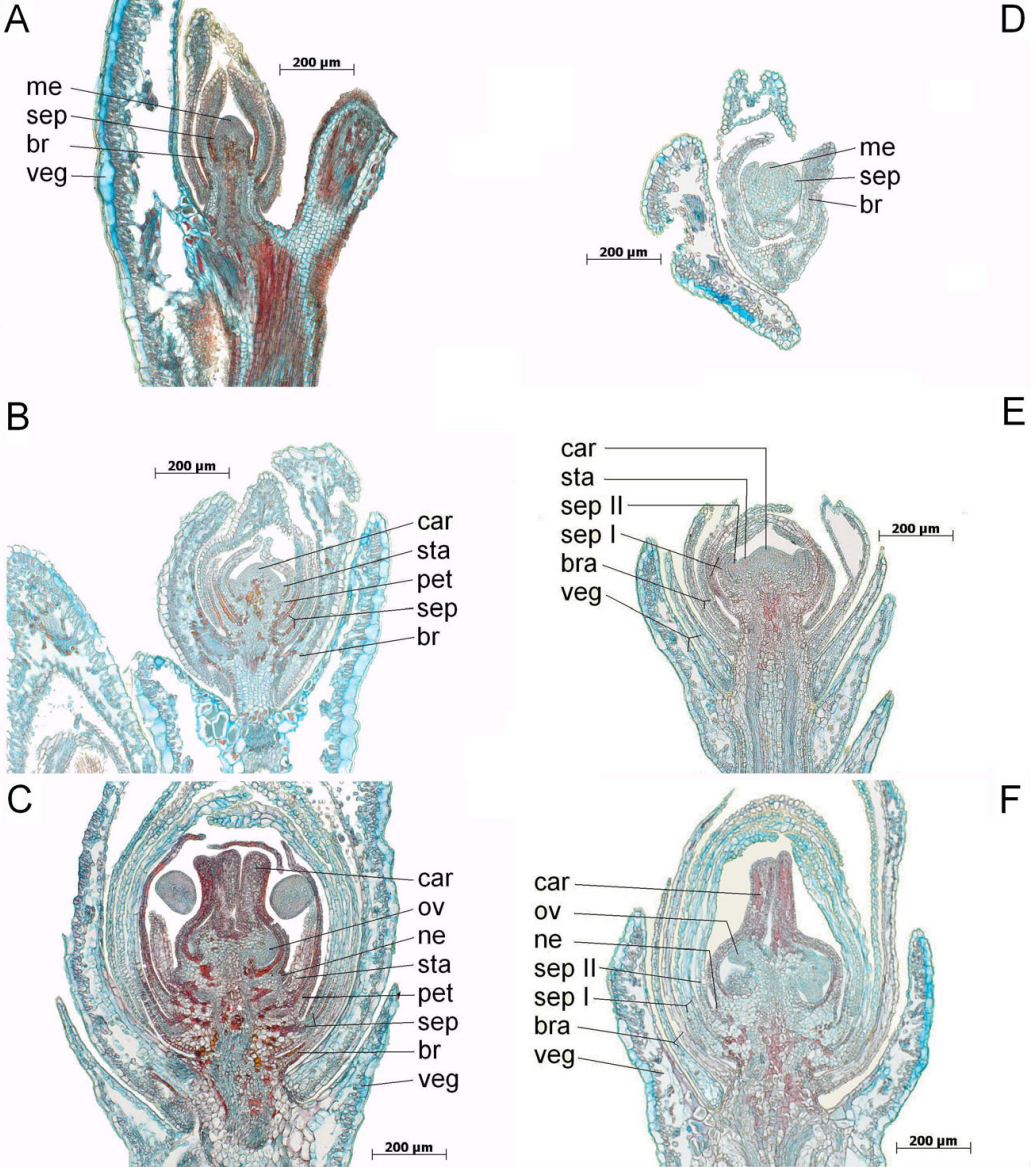
**Comparative investigation of *C. vulgaris *flower development**. Histological slices of 5 μm intervals were fixed in AFE and stained by FCA. Organs and tissues are labelled by veg (vegetative tissue), br (bracts), sep I or sep II (sepals, whorl no.), pet (petals, if available), sta (stamens, if available), ne (nectaroids), car (carpels), ov (ovules) and me (flower meristem), respectively. A-C: different stages of a wild-type inflorescence; D-E: different stages of a 'bud-flowering' inflorescence;

Flower organ development of the wild-type and the 'bud-flowering' type were investigated comparatively by histological analysis. Samples were derived from the uppermost part of shoots for which the initiation of flower development could undoubtedly be ascertained. Fig. 6 shows three equal stages of both the wild-type and the 'bud-flowering' type in parallel. Generative meristems of both flower types did not differ anatomically (Fig. 6AD). Both flower types also show the development of stamen primordia (Fig. 6BE). We classify these as such as a consequence of experiments in *A. thaliana *[25], since these authors describe initial nectary development during developmental stage 9. However, petal and stamen primordia already arise during the developmental stage 5 [26]. Therefore, nectary primordia in *C. vulgari*s seem not to develop until carpel formation. When the carpels are clearly recognizable as such (Fig 6CF), the comparison of wild-type and 'bud-flowering' types reveals there is no residual indication of former stamen formation in the latter phenotype.

**Figure 7:**
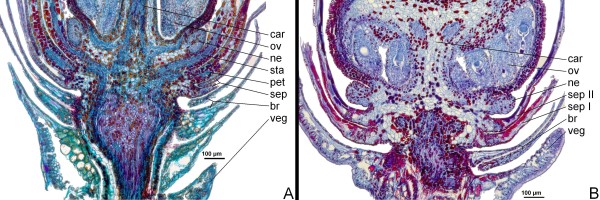
**Mature flowers of *C. vulgaris***. Histological slices of 8 μm intervals were fixed in Bouin-Allen's compound and stained by SGL. Organs and tissues are labelled by veg (vegetative tissue), br (bracts), sep I or sep II (sepals, whorl no.), pet (petals, if available), sta (stamens, if available), ne (nectaroids), car (carpels), ov (ovules) and me (flower meristem), respectively. A: wild-type; B: 'bud-flowering';

Interestingly, petal and sepal tissues are differently stained in the wild-type (Fig. 6C) but both whorls of petaloid sepal organs in the 'bud-flowering' type display the same staining pattern (Fig. 6F). Furthermore, in the wild-type, petals and stamens show a comparable staining pattern and petals consist of an increased amount of cell layers if compared to the petaloid sepals of the wild-type and the 'bud-flowering' type. This becomes even more obvious in opened, mature flowers of each type, using SGL instead of FCA staining (Fig. 7).

### Estimation of the genome size

The genome size of *C. vulgaris *was estimated by laser-based flowcytometry since the knowledge of this parameter is essential for future genetic applications. We compared seven wild-type, two 'bud-flowering', one 'filled' and one 'multi-bracteate' genotype from different countries (Table 1). Three to six replications of each sample led to an overall average genome size of 1.1799 +/- 0.0028 pg/2C (mean +/- standard error, n = 50). According to the equation given by [27], from this the total DNA length of *C. vulgaris *can be calculated to be approximately 1,154 Mbp.

**Table 1:** Flow cytometric estimation of the absolute DNA content of *C. vulgaris*.

Flower type	Denomination	Origin	pg DNA/2C	n
wild-type	*Löhnstein*	Germany	1.16 +/- 0.006	4

wild-type	*Niederohe*	Germany	1.17 +/- 0.008	4

wild-type	*San Remo*	Italy	1.20 +/- 0.006	5

wild-type	*Kvam*	Norway	1.19 +/- 0.015	4

wild-type	'Long White'	The Netherlands	1.18 +/- 0.011	6

wild-type	'Multicolor'	USA	1.18 +/- 0.016	5

wild-type	'Silver Knight'	UK	1.18 +/- 0.011	5

'bud-flowering'	'Karla'	Germany	1.17+/- 0.014	5

'bud-flowering'	'Sandhammeren'	Sweden	1.20 +/- 0.018	3

'filled'	'Radnor'	UK	1.20 +/- 0.018	4

'multi-bracteate'	'Perestroijka'	Germany	1.15 +/- 0.015	5

The table indicates the flower type, the denomination of the genotype or variety, the country of origin (if known) and the amount of measured replicates n. Genotypes in italics are samples collected in the wild.

## Discussion

The vague and differing descriptions of the flower anatomy ([5,6,8,11]) of *C. vulgaris *necessitated more in-depth investigations regarding the flower organ identity. We combined different microscopic (e.g. SEM) and molecular analyses (qRT-PCR), since both approaches are complementary (see, e.g. [12] and references therein). As a result from the indications received from these analyses, we were able to appoint the organ numbers for *C. vulgaris *wild-type and 'bud-flowering' phenotypes as summed up in the given floral formulas.

In wild-type flowers sepals and petals are morphologically clearly distinguishable. In contrast, regarding the 'bud-flowering' type, our anatomical analyses revealed that whorl II organs are macroscopically indistinguishable from the whorl I organs. In both flower types the uppermost green leaves have been identified as bracts, since they differ morphologically from both, sepals as well as leaves.

These morphological and anatomical data were supported by our gene expression analyzes. We detected expression of *CvAP3*, a class B-like MADS-box gene, mainly in the inner perianth organs of the wild-type phenotype. According to the classical 'ABCDE model' and its modifications, we anticipated expression of the *AP3/DEF*-like gene to be restricted to whorls II and whorls III [13]. In contrast, no difference of expression was observed between the whorl I and II organs in the 'bud-flowering' type, which supports our morphological (SEM) data suggesting an additional whorl of petaloid sepals and the coincidental loss of petals in this flowering type. Thus, differential expression of *CvAP3 *consistently reflects changes and similarities in the morphology of whorl I and whorl II flower organs in the wild-type as well as in the 'bud-flowering' type. However, these differences are of a quantitative and not of a qualitative nature. The gradual decrease of *AP3/DEF*-like gene expression between petals and sepals that we reported for one of three genotypes tested is already known from other *Ericales *(*Impatiens hawkeri*, [20]).

The expression pattern of *CvSEP1*, a class E-like MADS-box gene, reflects the expectations resulting from experiments in model organisms; namely, expression of *SEP1/2*-like genes was expectable for whorls II-IV, although 'expression in sepals is common but not universal' ([24], page 431). In *C. vulgaris *wild-type flowers, this expression was consistently reproduced expect for the variety 'Roter Oktober' that showed a surprisingly high and increased mRNA amount in whorl I. In 'bud-flowering' phenotypes, the petal whorl is presumably transformed into sepaloid sepals and thus, expression of *CvSEP1 *is lower, but comparable between whorl I and II.

Regarding the identification of bracts, our expression analyses also confirmed the morphological argumentation. On the one hand, the higher expression of both *CvAP3 *and *CvSEP1 *in these organs indicates a clear difference to leaves, especially for *CvSEP1*. On the other hand, besides the clear morphological dissimilarity, expression of *CvSEP1 *was obviously repressed in these uppermost green leaves compared to the sepals. Therefore, we identified these leaves as bracts. Again, this result is in line with another *Ericales *(*Marcgravia umbellata*), in which a *DEF*-like gene was shown to be expressed at low levels in bracteoles/sepals compared to petals or stamens of the same species [20].

Expression of *CvAP3 *and *CvSEP1 *was detected and confirmed in leaves independent of tissue age for both flowering-related genes. Expression of floral organ identity genes in non-floral tissues is already known from other species. In *Gerbera*, the *SEP1/2 *gene *GhGRCD2 *is expressed in vegetative tissues and *SEP3*, usually restricted to the inner three whorls, is described to be expressed in vegetative tissues in more than one species, too ([24] and references therein). Likewise, in Rose expression of the *AP3*-like gene *MASAKO euB3 *was detected in vegetative tissue [28].

Regarding our results, it has to be borne in mind that, according to the floral quartet model, floral organ identity genes concertedly regulate the organ identity [29]. Petal identity in eudicots, for example, is usually based on the simultaneous occurrence of *AP3/DEF*-like, *PI*-like and *SEP3*-like gene products, since these are all required for establishing full petal identity. Furthermore, epidermal cell shape is known to be controlled by MYB transcription factors, which themselves are, in turn, under control of class B-like genes [30,31]. It was shown recently, that *SEP3 *expression in *A. thaliana *is spatially distinguishable between ab- and adaxial petal sides [32] and hence, may be at least partially responsible for cell surface shaping as it was already known for other SEP-like genes [33]. Thus, our analyses necessarily remain incomplete and comprehensive results require substantially more laboratory and phenotyping experiments. Nevertheless, we presumably were able to differentiate all organs in question by expression analyses of just two putative MADS-Box transcription factors.

The wild-type flower of *C. vulgaris *is synoecious, while the final 'bud-flowering' flower is unisexually female due to a total loss of once initiated stamens. This is in line with the claim, that every unisexual flower that has been investigated until now showed a certain degree of initial hermaphroditic characteristics [34]. Whether the change of organ identity in the perianth and the loss of stamen are necessarily linked remains to be analysed.

Within the *Ericaceae*, the genome size is only known in seven *Vaccinium *species [35]. Here, the nuclear DNA content ranged from 1.20 - 7.20 pg/2C. Knowledge of the genome size is an essential prerequisite for prospective genomic applications in this species including mapping and genome walking for isolation of putative genes responsible for the 'bud-flowering' genotype. Although the measured value of 1.18 pg/2C is low, it is still approx. four times higher than in *Arabidopsis *(0.3 pg/2C, [36]. Nevertheless, it facilitates the construction of a BAC (Bacterial Artificial Chromosome) library and subsequent map-based cloning.

## Conclusions

Our study presents a first step towards the analyses of flower organ identity and their modifications in the ornamental crop *C. vulgaris*. We confirmed the identity of petals, sepals and bracts in wild-type as well as in the 'bud-flowering' phenotypes.

The simultaneous degeneration of stamens and the conversion of petals to sepals in the 'bud-flowering' type cannot be explained by modifications of the 'ABCDE'-model. Neither can apparent candidate genes be deduced from comparison with other plant species so far.

Further investigations should include additional cloning of further floral organ identity gene homologs as well as studies of their expression in all floral organs of the relevant flower types. Since a comprehensive understanding of the genetics of the 'bud-flowering' phenotype is a prerequisite for future breeding of this economically important ornamental crop, mapping of this trait with subsequent map-based cloning will be the next step to identify candidate genes, since the relatively small genome size of *C. vulgaris *allows efficient construction of a BAC library.

## Methods

### Histological Techniques and Microscopy

Tissues were fixed for at least 24 h in AFE (10.4 : 1 : 1 96% ethanol : formalin : acetic acid) or for max. 4 h in Bouin-Allen's compound (14 : 5: 1 picronitric acid : formol : acetic acid + 1.48% (w/v) CrO_3_), dehydrated by an increasing ethanol/isopropanol series, infiltrated and embedded in paraffin under low air pressure conditions, and sectioned at varying μm-intervals using a Leica RM2155 microtome. The sections were stained with either FCA (fuchsin CI42520, chryosidine CI11270, astral blue CI48048; staining: 5 min; washing: H_2_O, 10 sec; 2× washing: 30% ethanol, 30 sec; differentiation: 70% ethanol, 30 sec; 2× washing: 30% ethanol) or SGL (safranine CI 50240, pyoctanin blue CI 42535, acid green CI 42095; staining I: safranine, 60 min; washing: H_2_O, 2 min; staining II: pyoctanin blue, 3 min; washing: H_2_O, 5 min; washing: isopropanol, 1 min: staining III: acid green, 1 min; 4× washing: isopropanol, clove oil, isopropanol, terpineol (each 1 min)) and photographed by a Zeiss Axio Imager.A1. The macroscopical analysis of the flower morphology was performed using a Leica Wild MZ3 stereo microscope. The following varieties were used: 'Wink 1-2006', 'Wink 2-2006' ('bud-flowering'), 'Roter Oktober', *SanRemo *(wild-type).

### Scanning Electron Microscopy

Samples were fixed over night in FAEG (ethanol (65%), acetic acid (5%), 37% formaldehyde (3.2%), 50% glutaraldehyde (0.2%), Tween-20 (0.1%), H_2_O) and dehydrated by an ethanol series: 15 min 80% ethanol, 15 min 90% ethanol, 15 min 96% ethanol, 3 × 20 min 100% ethanol. The samples were then transferred to 100% acetone (3 × 20 min) and subsequently critical point dried using liquid CO_2 _in an EMITECH K850. The leaves were mounted on Leit-Tabs and gold-coated (sputter-coater: EMITECH K500). Observations of the abaxial and adaxial sides of the perianth organs of each three genotypes were performed using a Philips XL30 ESEM (at the Institute of Systematic Zoology and Evolutionary Biology, University of Jena) with a voltage of 10 kV.

The following varieties were investigated: 'Battle of Arnhem', 'Karmina', 'Roter Oktober', 'Silver Knight' (all wild-type) and 'Adrie', 'Annegret', 'Nicole', 'Wink 2-2006' (all 'bud-flowering'). Selected, representative images are shown in Figs. 2 and 3.

### Cloning of MADS-box genes

Total RNA of wild-type *C. vulgaris *'Roter Oktober' flower buds was isolated using a modified protocol of the RNeasy Plant Mini Kit ([37], Qiagen) and subsequently reverse transcribed to first strand cDNA (Reverse Transcription System, Promega) using a standard oligo(dT) primer: GACTCGAGTCGACATCTG(T)_14_. 3'-RACE-PCR [38] was performed using a degenerated 5'-B-gene-MADS-box-specific primer (5'-TSAAGAAAGCWWARGAGCTYWCCG) and the corresponding 3'-nested primer derived from the oligo(dT) primer. Amplified fragments of appropriate size were gel-extracted (Nucleo Spin Extract II kit, Macherey-Nagel), ligated into the pDRIVE vector and transformed into EZ cells (Qiagen PCR Cloning plus kit) by heat-shock. Cells were plated on standard LB/Amp/IPTG/X-Gal plates. Plasmid DNA from positive clones (blue/white selection plus colony-PCR testing) was extracted (E.Z.N.A. Plasmid mini kit II, Omega bio-tek) and sequenced (MWG Biotech AG, JenaGen GmbH, AGOWA GmbH).

Alignments of derived sequences were accomplished by ClustalW2 [39] or T-Coffee [40]. BLASTx 2.2.19+ [41] and BLASTn 2.2.19+ [42] were used to check the *C. vulgaris *sequences for matching hits at the protein or nucleotide level. Cloned genes were named using the abbreviation of the species name and the gene class, respectively, and uploaded to the GenBank database via Sequin.

Verification of gene identity was additionally performed by motif analysis within alignments on protein level (Additional Files 5 and 6). Phylogenetic data analysis was performed using GeneDoc alignments [43] and Paup 4.0 [44].

### Expression analysis (qRT-PCR)

Total RNA of the varieties under investigation was isolated using the original manufacturer's protocol of the Invisorb Spin Plant RNA Mini Kit. cDNA was reverse transcribed using the original protocol of the QuantiTect Reverse Transcription Kit (Qiagen). To provide better sample comparability, isolation and reverse transcription was performed simultaneously for all samples. qRT-PCR primers (Table 2) were designed to target the *AP3/DEF*- and *SEP1/2*-like genes using Primer3Plus [45]. The partial sequence of *C. vulgaris *18S rRNA [GenBank:AF419797] was used to design normalizing primers. PCR reactions (3 independent runs with each 3 technical replicates of three 'bud-flowering' ('Annegret', 'Nicole', 'Wink 2-2006') and three wild-type ('Karmina', 'Battle of Arnhem', 'Roter Oktober') genotypes) were performed with 0.5 ng cDNA (quantified via Qubit Fluorometer (Invitrogen)) on a Stratagene MX3000P thermocycler (qPCR MxPro v4.01) using the Absolute qPCR SYBR green ROX mix (ABgene). Gene expression analysis was normalized vs. *C. vulgaris *18S rRNA. ΔΔCt, i.e. the fold change was calculated according to *Ratio *= 2^-ΔΔCt ^[46], whereas the mean ΔCt of the vegetative tissue was subtracted from the normalized ΔCt-values of bracts, sepals and petals, respectively. Prior to realtime PCR experiments, primer combinations were tested for their optimum concentration, the prerequisite of PCR-product-free non-template controls and for comparable amplification efficiencies according to common methods [47,48]. The qRT-PCR products were additionally verified for length (electrophoretic separation) and sequence (AGOWA GmbH) identity with the predicted amplicons derived from different tissues and genotypes.

**Table 2:** qRT-PCR primer sequences designed to amplify products < 200 bp.

Target sequence	Primer Sequence	Product size [bp]
18S rRNA [GenBank:AF419797]	Forward: GGGATGAGCGGATGTTACTT Reverse: CCCTTCCGTCAATTCCTTTA	116

*CvAP3* [GenBank:GQ202026]	Forward: TCGACGAGCTGAATAGTCTTGA Reverse: TCGACTAGCCCATAGTGTGGAT	190

*CvSEP1* [GenBank:GQ202027]	forward: AGCATCATCCTCAATCCCAG Reverse: GATCATTCCGCTCACGTTTT	143

### Estimation of nuclear genome size by flow cytometry

Fresh young foliage from samples and internal reference standards (0.5 cm^2 ^each) were co-chopped with a sharp razor blade in a Petri dish containing 500 μL nuclei isolation buffer according to [49], supplemented with 1% polyvinylepyrrolidone 25, 0.1% Triton X-100, 50 μg/ml RNAse and 50 μg/ml propidium iodide, incubated for at least 30 sec and filtered through a 35 μm mesh. The relative fluorescence intensities of stained nuclei were measured on a FACStar^PLUS ^(BD Biosciences, San Jose, CA, USA) equipped with an INNOVA 90-C argon laser (Coherent, Santa Clara, CA, USA). Propidium iodide was excited at 514 nm and measured in FL1 channel using a 630 nm band-pass filter. At least three plants of each *C. vulgaris *sample were used for absolute DNA content estimation together with *Glycine max *(L.) Merr. convar. max var. max ('Cina 5202', 2C = 2.23 pg; Genebank Gatersleben, accession number: SOJA 392) as an internal standard. The nuclear DNA amount of the standard was determined based on the value of 0.32 pg/2C for *Arabidopsis thaliana *'Columbia' [50]. Usually 10,000 nuclei per sample were analyzed. The absolute DNA amounts of the samples were calculated based on the values of the G1 peak means. ANOVA HSD Post-hoc test for unequal N, which is a modification of the Tukey HSD test, was used to determine significant differences between group means (p = 0.05).

## List of Abbreviations

A: Androecium; AP: APETALA; BAC: Bacterial Artificial Chromosome; Ca: Calyx; Co: Corolla; G: Gynoecium; MADS: mini-chromosome maintance1, Agamous, Deficiens, serum response factor; RACE: rapid amplification of cDNA ends; SEM: Scanning Electron Microscopy; SEP: SEPALLATA.

## Authors' contributions

TB carried out the establishment of all molecular methods and the experiments, performed the sequence alignments and all other genetic and molecular data analysis, captured the macroscopic, histological and SEM images and drafted the manuscript. KE participated in the qRT-PCR experiments. KK established the histological methods for *C. vulgaris *and carried out the complete histological analyses. JF established and carried out the flowcytometric methods and experiments. AH participated in the experimental design and critically revised the manuscript. All authors read and approved this final manuscript version.

## Supplementary Material

Additional file 1 **Alignment of *CvAP3 *with AP3/DEF-like gene sequences**. GeneDoc Document alignment file including the accession numbers of the sequences aligned. Click here for file

Additional file 2 **Alignment of *CvSEP1 *with SEP1/2-like gene sequences**. GeneDoc Document alignment file including the accession numbers of the sequences aligned. Click here for file

Additional file 3 **Unrooted consensus phylogram of *CvAP3 *alignment of Additional File **1**as computed by PaupUp**. Parameters used: best-fit model GTR+I+G selected by AICc (corrected Akaike Information Crite-rion, PaupUp), base frequencies 0.3048 (A), 0.2147 (C), 0.2521 (G), 0.2284 (T), burnin = 8500. Internal edge labels are equivalent to posterior probability values. Click here for file

Additional file 4 **Unrooted phylogram of *CvSEP1 *alignment of Additional File **2, **as computed by PaupUp**. Parameters used: Best-fit model HKY+I+G selected by AICc (PaupUp), base frequencies 0.3364 (A), 0.2244 (C), 0.2278 (G), 0.2113 (T), Ti/tv ratio = 1.3025, Burnin = 700. Internal edge labels are equivalent to posterior probability values. Click here for file

Additional file 5 **Aligned AP3/DEF-like protein sequences**. Translated protein sequences, aligned, gene-identifying motifs are highlighted. Click here for file

Additional file 6 **Aligned SEP1/2-like protein sequences**. Translated protein sequences, aligned, gene-identifying motifs are highlighted. Click here for file
