# Supplementary figures and images for: 'Who's who' in two different flower types of Calluna vulgaris (Ericaceae): morphological and molecular analyses of flower organ identity
Source: BMC Plant Biol. 2009 Dec 14;9:148. doi: 10.1186/1471-2229-9-148 (PMC2803492; doi:10.1186/1471-2229-9-148)

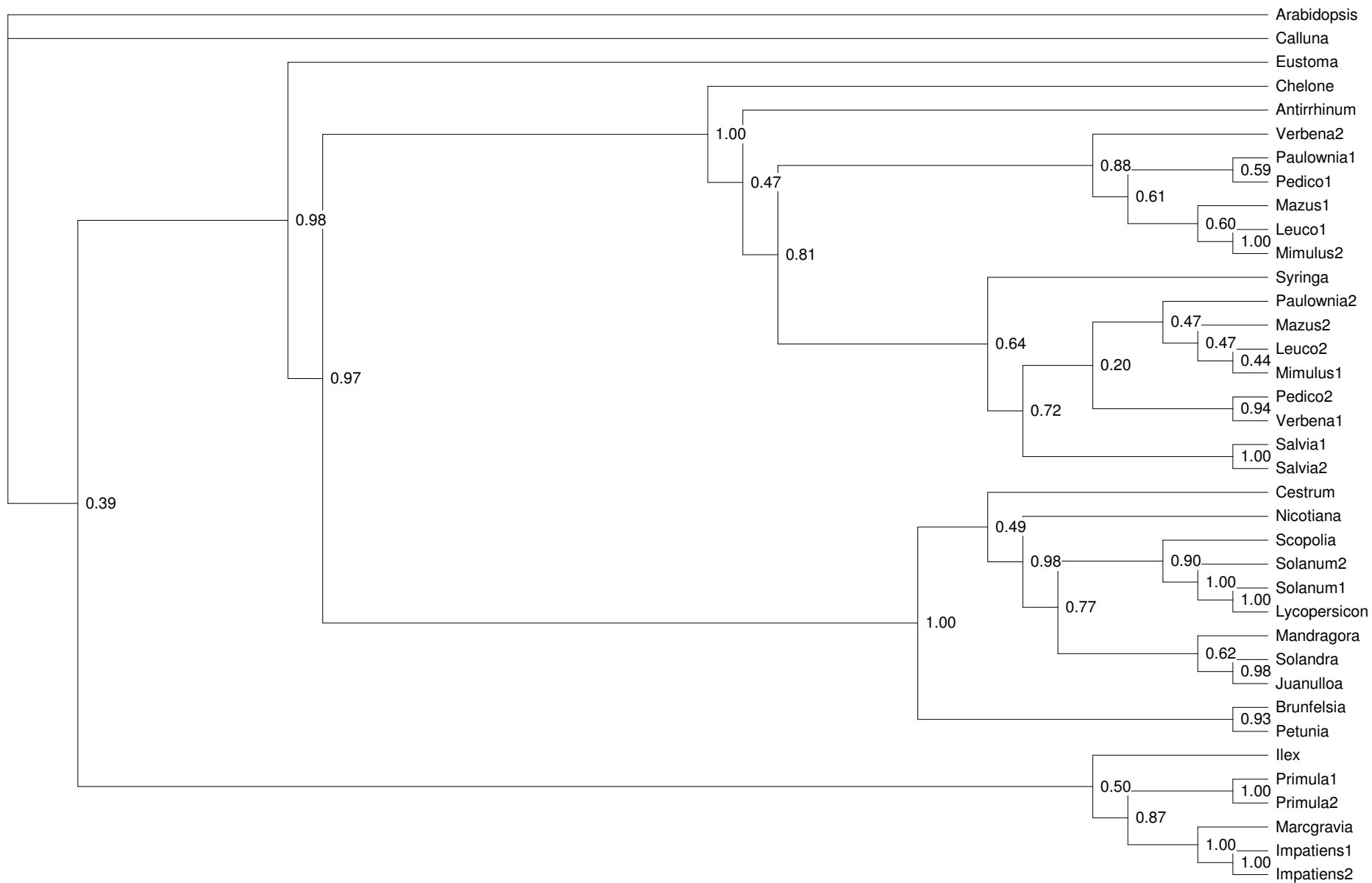

Supplement: Additional file 3 — Unrooted consensus phylogram of CvAP3 alignment of Additional File 1as computed by PaupUp. Parameters used: best-fit model GTR+I+G selected by AICc (corrected Akaike Information Crite-rion, PaupUp), base frequencies 0.3048 (A), 0.2147 (C), 0.2521 (G), 0.2284 (T), burnin = 8500. Internal edge labels are equivalent to posterior probability values. [file 1471-2229-9-148-S3.PDF]

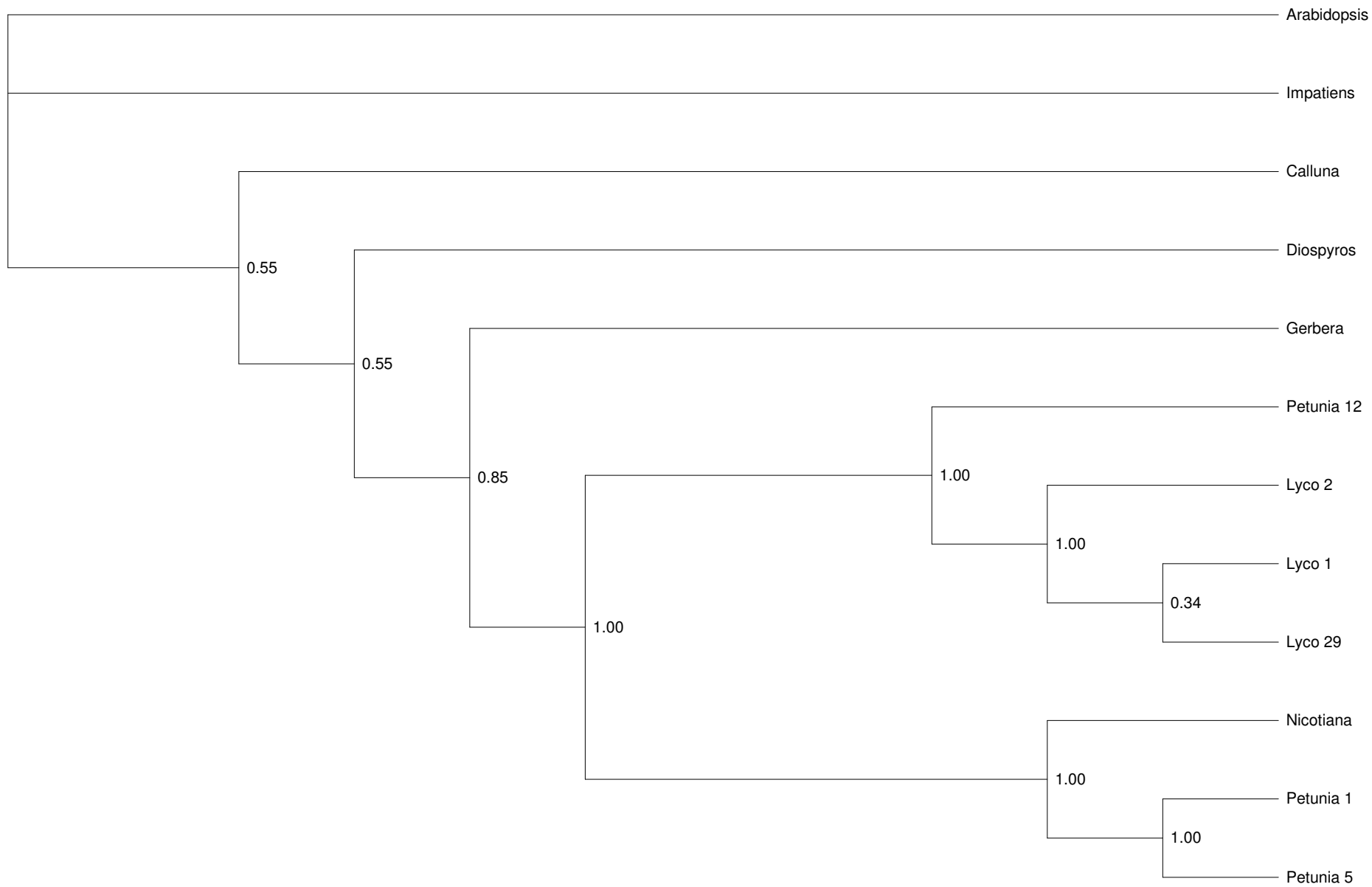

Supplement: Additional file 4 — Unrooted phylogram of CvSEP1 alignment of Additional File 2, as computed by PaupUp. Parameters used: Best-fit model HKY+I+G selected by AICc (PaupUp), base frequencies 0.3364 (A), 0.2244 (C), 0.2278 (G), 0.2113 (T), Ti/tv ratio = 1.3025, Burnin = 700. Internal edge labels are equivalent to posterior probability values. [file 1471-2229-9-148-S4.PDF]
