# Supplementary material for: 'Who's who' in two different flower types of Calluna vulgaris (Ericaceae): morphological and molecular analyses of flower organ identity
Source: BMC Plant Biol. 2009 Dec 14;9:148. doi: 10.1186/1471-2229-9-148 (PMC2803492; doi:10.1186/1471-2229-9-148)
Supplement: Additional file 5 — Aligned AP3/DEF-like protein sequences. Translated protein sequences, aligned, gene-identifying motifs are highlighted. [file 1471-2229-9-148-S5.PDF]

```
                                                                                   PI Motif-derived        EuAP3
                 *       160         *       180         *       200         *       220         *       240         *       260
Paulownia1 : QIETSKKKLRNVEEIHRNLVLEIDAR-QEDPHYG-----LVENEGD---------YNSVLGFPNGG-PRIIALRLPP-NHHHHHHHH--------PSLHSGGAA-----SDLTTFALLE- : 214
Pedicu1    : QIETGKKKLRNVEEIHRNLVLEFDAR-EEDPHYG-----LVENEGD---------YNSVLGYPNGG-SRIIALRLP---HHH-------------PGLHSGGAA-----SDLTTFALLE- : 219
Leuco1     : RIETSKKKLRNVEEIHRSLVLEFDAR-QEDPHFG-----LVENEGD---------YNSVLGFPHGG-PRIIALHLPS-NHQHHHNHHH-------PGLHSGGAGG----SDLTTFALLE- : 228
Chelone    : QIETSKKKVRNVEEIHRSLVLEFDAT-----HYG-----LIENEGD---------YNSILGFPNRG-QRIIALRLP--THPDDHH----------PSLHSGGGG-----SDLTTFALLD- : 219
Syringa    : QIETSKKKLRNVEEIHRNILLEFDAR-QEDPQYG-----LVDNEGD---------YNSVLGFPNGG-PRIIALRLPS-NHH--------------PNLHSGGG------SDLTTFALLE- : 219
Verbena1   : QIDTSKKKLRNVEEIHKNLVLEFDAR-QEDPHYG-----LVENEGD---------YNSVLGFPNGG-PRIIALRLPP-NHHLNHHPN---------HLHGGGG------SDLTTFHLLDQ : 225
Scopolia   : QIETFKKKVRNVEEIHKNLLLEFDAR-EEDP-YGG----LVEQEGD---------YNFMLGFPNG--DHILTLRLQPNNHHHHHHHH---------HLHSGGG------SDITTFGLA-- : 222
Mandragora : QIETYKKKVRNVEEIHRNLLLEFDAR-QEDP-YG-----LVEQEGD---------YTSVLGFQNGG-PRILALRIQPNN-----------------HLHSGGG------SDITNFGLA-- : 214
Solandra   : QIETYKKKVRNVEEIHRNLLLEFDAR-QEDP-YG-----LVEQEGD---------YNSVLGFPNGV-PRILALRLQPNNHHHHHH-----------HLHSGGG------SDITTFALA-- : 220
Juanulloa  : QIETYKKKVRNVEEIHRNLLLEFDAR-QEDP-YG-----LVEQEGD---------YNSVLGFPNGG-SHILALRLQPNNHNHH-------------HLHSGGG------SDITTFALA-- : 218
Cestrum    : QIETYKKKVRNVEEIHRNLLLEFDAR-QEDP-YG-----LVEQEGD---------YNSVLGFQNGG-HRILALRLQP-NHH---------------HLQSGGG------SDITTFALG-- : 215
Brunfelsia : QIETCKKKVRNVEEIHRNLLLEFDAR-QEDP-YG-----LVEQEGD---------YNSVLGFPNGSGHRILALRLQP-NHHHQPNHHHHHHH----HLHSGGG------SDITTFALLE- : 228
Ilex       : QVDTYKKKLRNVEEIHRNLLHEFDLR-DEDPHYG-----LVDNGGD---------YDSVLGFPNGG-PHILALRLQPNQ----------------PNPHSGG-------SDLTTYALLE- : 200
Salvia1    : RIDTSRKKLRNVEEIHRGLVLQFDAR-QEDPHYG-----LVENEGD---------YNSMLGFPHGG-PRIIAVRLPPNNHHPHPHHHHHHHHHH-PSLHSGTGA-----SDLTTFALLE- : 242
Mazus2     : QIDTTKKKVRNVEEIHRGLVLEYEAR-HEDPHYG-----LVENEGDP--------YNSVLGFPSGG-PRIVALHLP--QHHHHHNHHHHHGG----SFHSGGA------SDLTTFALLE- : 237
Mimulus2   : RIETGKKKVRNVEEIHRNLVLEFEAR-QEDPHYG-----LVENEGD---------YNSFLGFAHGG-PRIVALHVPP-NHHHHHHH---------PSLHSGGGAG----SDLTTFALLDQ : 235
Eustoma    : QIETLKKKVKSANDIHRNLLLELDAR-QEDPHYG-----LVENAGD---------YHSLIGLPNGG-HHVLALCLQP-NP---------------PSFISGGG------SDLTTFALLD- : 226
Solanum1   : QIETYRKKVRNVEEIHRNLLLEFDAR-QEDP-YGG----LVEQEGD---------YNSVLGFPTGG-HHILALGLQP-NNNHHH------------HLHSGGG------SDITTFALG-- : 228
Lycopers   : QIETYRKKVRNVEEINRNLLLEFDAR-QEDP-YGG----LVEHDGD---------YNSMLGFPTGG-PRILDLRLQP-NNNYHN------------HLHSGGD------SDITTFALG-- : 228
Nicotiana  : QIDTYKKKVRNVEEIHRNLLLEFDAR-QEDP-YG-----LVEQEGD---------YNSVLGFPNGG-PRILALRLQP-NHQPNH------------HLHSGGG------SDITTFALA-- : 227
Petunia    : QIETFKKKVRNVEEIHRNLLLEFDAR-QEDP-YG-----LVEQEGD---------YNSVLGFPNGG-HRILALRLQP-NHHQPNHHH---------HLHSGGG------SDITTFALLE- : 231
Antirrhinu : QIDTSKKKVRNVEEIHRNLVLEFDAR-REDPHFG-----LVDNEGD---------YNSVLGFPNGG-PRIIALRL-PTNHH--------------PTLHSGGG------SDLTTFALLE- : 227
Primula1   : QIETSRKKVRNGEEVHRTLLLEFDAR-EEDPHYG-----LVDNGGD---------YDSVIGYTNEGEPRMLSLRLQP-NHH---------------NLASGGGG-----NGLTTYALL-- : 228
Primula2   : QIETSRKKVRNGEEVHRTLLLEFDAR-EEDPHYG-----LVDNGGD---------YDSVIGYTNEGEPRMLSLRLQP-NHH---------------NLASGGGG-----NGLTTYALL-- : 228
Impatiens1 : QIETHRKKLRNVEQIHRNLLQEFDVR-EED--VVQVECGVGGLMENMNGHGGGGEYVGGGFHGFGGRSSTTSPRIFAVRTPAPASGNQMMRRSSI-NLQSSTTAAGVVGSDLTTYALL-- : 259
Impatiens2 : QIEIHKKKLRNVEQTHRNLLQQFGFREEEETQYA-----LAENEGG---------WL--------------------------------------------------------------- : 188
Marcgravia : QIETFKKKLKNVEQIHRNLLQEFDLR-EDEPHYG-----LVDNGGE---------YAPFNGFTTRS-PRILAVRLQP-NQK---------------SLHSGVG------SDLTTYTLL-- : 209
Calluna    : QTDTMKKKVRNVEIIHRSLLHEFDAI-GEDPHYG-----LVDNGGY---------SNGARG--------LLALRLQP-NHHHNLHSLQANHH----NLHSGTG------SDLTTFALLE- : 198
Arabidopsi : QIETTKKKNKSQQDIQKNLIHELELR-AEDPHYG-----LVDNGGD---------YDSVLGYQIEG-SRAYALRFHQ-NHHHYYPNHG---------LHAPSA------SDIITFHLLE- : 232
```
