# Supplementary material for: 'Who's who' in two different flower types of Calluna vulgaris (Ericaceae): morphological and molecular analyses of flower organ identity
Source: BMC Plant Biol. 2009 Dec 14;9:148. doi: 10.1186/1471-2229-9-148 (PMC2803492; doi:10.1186/1471-2229-9-148)
Supplement: Additional file 6 — Aligned SEP1/2-like protein sequences. Translated protein sequences, aligned, gene-identifying motifs are highlighted. [file 1471-2229-9-148-S6.PDF]

```
                                                                  SEP I                             SEP II
                   100         *         120         *         140         *         160         *         180
Gerbera      : KLEEVYAENQAG----PSWAA--GEHHS-SYGQEHQHQHSQGFFQPLDCNSNLQI--GYNTV-DSSHIT-AS-T--NGQNLNGLIPGWML : 169
Lyco_1       : KMEEIYAENNMQ----QAWGG--GEQSL-NYGQ--QQHPQSQGFFQPLECNSSLQI--GYDPITTSSQIT-AV-T--NAQNVNGMIPGWML : 168
Lyco_2       : KMEEIYAENNMQ----QAWGG--GEQSL-NYGQ--QQHPQSQGFFQPLECNSSLQI--GYDPITTSSQIT-AV-T--NAQNVNGMIPGWML : 168
Lyco_29      : KMEEIYAENNMQ----QAWGG--GEQSL-NYGQ--QQHPQSQGFFQPLECNSSLQI--GYDPITTSSQIT-AV-T--NAQNVNGMIPGWML : 168
Petunia_1    : KLEQIYAENNIQ----QSWGG--GQQSG-AYSQ---Q-AQTQGFFQPLECNSTLQI--GYDPT-TSSQIT-AV-T--SGQNVNGIIPGWML : 167
Petunia_5    : KLEQIYAENNIQ----QSWGG--GEQSG-AYGQ---QHAQTQGFFQPLECNSTLQI--GYDPA-TSSQIT-AV-T--SGQNVNGIIPGWML : 168
Petunia_12   : KLEEIYAENSLQ----QSWGG--GEQSV-TYGH--QHNAQSQGFFQPLECNSTLQI--GYNPITTSRQIT-AV-T--NAQNVNGMVPGWML : 168
Nicotiana    : KLEEIYAENNLQ----QSWGGG-GEQSG-AYSQ---QHPQTQGFFQPLECNSTLQI--GYDPA-SSSQIT-GV-T--SGQNINGIVPGWML : 154
Calluna      : KLDDIYRENHLQ----STWAC--GEQSN-TFGNP-QHHPQSQGFFQPLECNPNLQI--GYNPQ-VSNQLTPAATTHGQGQNVSGMIPGWML : 174
Impatiens    : RLLESS----------FNWMQ--NGQHV-DYSGP-AVQPN-DELFHPLECEPTLQMAMGYQTHHDPTSVE-AA-G--AGPSMNNYFPGWLS : 165
Diospyros    : KLDEIYRENQLQ----SSWGGGGEQGNSSFNHH-HHHPHSQAFFHPFDCNPTLQI--GYPEV-SNQMGT-AA-T--HEQNMNGLVPEWML : 171
Arabidopsi   : KLDDMIGVRSHHMGGGGGWEG--GEQNV-TYAH---HQAQSQGLYQPLECNPTLQM--GYDNPVCSEQIT-AT-TQAQAQQGNGYIPGWML : 173
```
